# Supplementary material for: Conditional RNA interference in mammalian cells via RNA transactivation
Source: Nat Commun. 2024 Aug 10;15:6855. doi: 10.1038/s41467-024-50600-w (PMC11316766; doi:10.1038/s41467-024-50600-w)
Supplement: Supplementary file 1 — Supplementary Information [file 41467_2024_50600_MOESM1_ESM.pdf]

Supplementary Information for

Conditional RNA Interference in Mammalian Cells via RNA Transactivation

Yu Zhou[1,2 †], *Peike Sheng*[1,3,4 †], *Jiayi Li*[5,6], *Yudan Li*[6,7], *Mingyi Xie*[1,3,4]*, *Alexander A. Green*[5,6,7]*

[1]UF Center for NeuroGenetics (CNG), Gainesville, FL, USA

[2]Department of Molecular Genetics and Microbiology (MGM), University of Florida, Gainesville, FL

[3]Department of Biochemistry and Molecular Biology, College of Medicine (COM), University of Florida, Gainesville, FL, USA

[4]UF Health Cancer Center, Gainesville, FL, USA

[5]Department of Biomedical Engineering, Boston University, Boston, MA 02215, USA

[6]Biological Design Center, Boston University, Boston, MA 02215, USA

[7]Molecular Biology, Cell Biology and Biochemistry Program, Boston University, Boston, MA 02215, USA

[†]These authors contributed equally

*Correspondence: aagreen@bu.edu, mingyi.xie@ufl.edu

Supplementary Note 1 | NUPACK design script used for generating ORIENTRs.

material = RNA
temperature = 37
trials = 10
structure Hpin = U18 D13(U1 D8(U2 D10 U8 U2) U1) U1 D9(U2 D8 U23 U2) U30
structure Trigger = U3 D8 U6 U3 U37 U6
structure Complex = U3 D37(U2 D10 U8 U12 D6(U1 D4(U2 D9(U2 D8 U23 U2) U2) U1) U17 + U3 D8 U6 U3) U6

domain preG = GGG
domain stem_loop = N30
domain trig = N37
domain Flank5 = N24
domain guide = ACCGUGUUGCUACAGCUAUAAG
domain Loop = UAGUGAAAUAUAUAUUAAA
domain passenger = CAUAUAGCUGAUGCAACACGGA
domain Flank3 = N12GAUACAGCAACUUUUUU
domain CapHp = N22
domain spacer = N3
domain pol_III_term = UUUUUU

Hpin.seq = preG trig stem_loop Flank5 guide Loop passenger Flank3
Trigger.seq = preG CapHp spacer trig* pol_III_term
Complex.seq = preG trig stem_loop Flank5 guide Loop passenger Flank3 preG CapHp spacer trig* pol_III_term

Hpin.stop = 10
Trigger.stop = 10
Complex.stop = 10
prevent = AAAA, CCCC, GGGG, UUUUU, KKKKKK, MMMMMMM, RRRRRRR, SSSSSSS, WWWWWWW, YYYYYYY

#Positive priRNA =
GAACUGACAUACUUGUUCCACUCACCGUGUUGCUACAGCUAUAAGUAGUGAAAUAUAUAUUAAACAUAUAGCU
GAUGCAACACGGAUAGUGUGACAGGGAUACAGCAACUUUUUU

Supplementary Figures

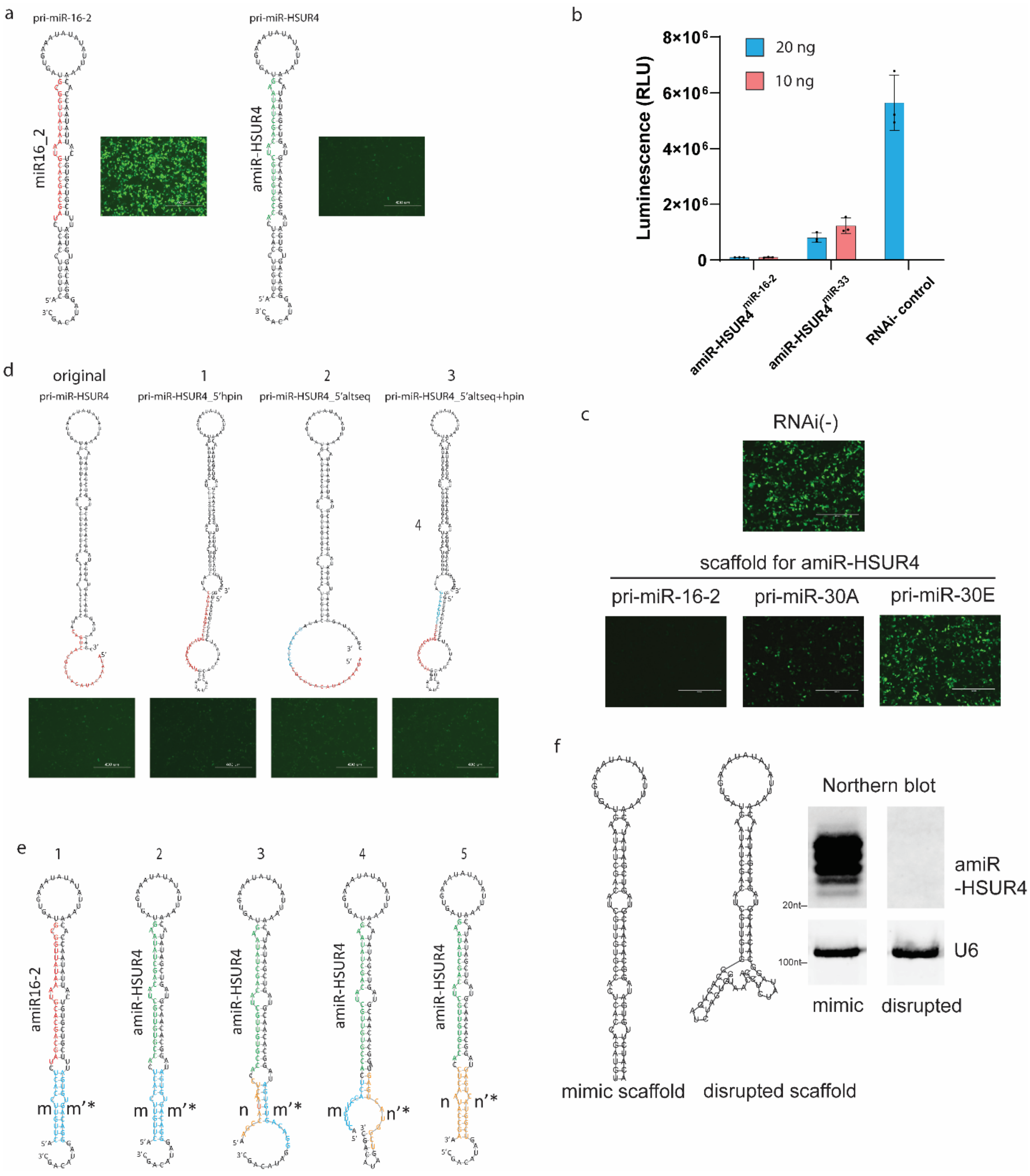

Figure S1 | Study of different pri-miRNA scaffolds. a, pri-miR-16-2 (left) and pri-miR-HSUR4 (right). In pri-miR-HSUR4, the sequence for miR16-2 was swapped with amiR-HSUR4, a viral microRNA sequence, and the secondary structure was preserved. A GFP reporter mRNA harboring a miR-HSUR4

target site is silenced by pri-miR-HSUR4 but not by pri-miR-16-2. n = 3 biological replicates. Scale bar represents 400 μm. **b**, Dual luciferase assay to characterize knockdown efficiency from the pri-miR-16-2 scaffold and the pri-miR-33 scaffold using amiR-HSUR4-5p. The pri-miRNA scaffolds were transcribed from a CMV promoter and produced amiR-HSUR4 to knockdown a luciferase reporter. Blue: 20 ng amiRNA plasmid versus 20 ng reporter; Pink: 10 ng amiRNA plasmid versus 20 ng reporter plasmid. n = 3 replicates (cells from same passage number were used for 3 replicates), bars represent the mean ± s.d. **c**, pri-miRNA scaffold comparison between pri-miR-16-2, pri-miR-30A and pri-miR-30E using amiR-HSUR4-5p with GFP as reporter. n = 3 biological replicates. Scale bar represents 400 μm. **d**, The original scaffold 5′ flanking sequence (red) upstream of the basal stem was modified in terms of sequence and structure by: (1) structurally sequestering the upstream sequence with base-pairing in cis, (2) inserting an 8-nt sequence (blue) between the flanking sequence and the basal stem, and (3) inserting the 8-nt sequence (blue) while introducing a 5′ base-pairing structure in cis. n = 3 biological replicates. Scale bar represents 400 μm. **e**, Nucleotide-level secondary structures of pri-miRNA 16-2 variants used in Fig. 1b. **f**, The pri-miR-16-2 scaffold producing amiR-HSUR4-5p was modified in the basal stem region to create a mimic scaffold that preserves the structure and a disrupted scaffold with a disrupted basal stem. The northern blot was performed on amiR-HSUR4 and U6 RNA. n = 3 biological replicates.

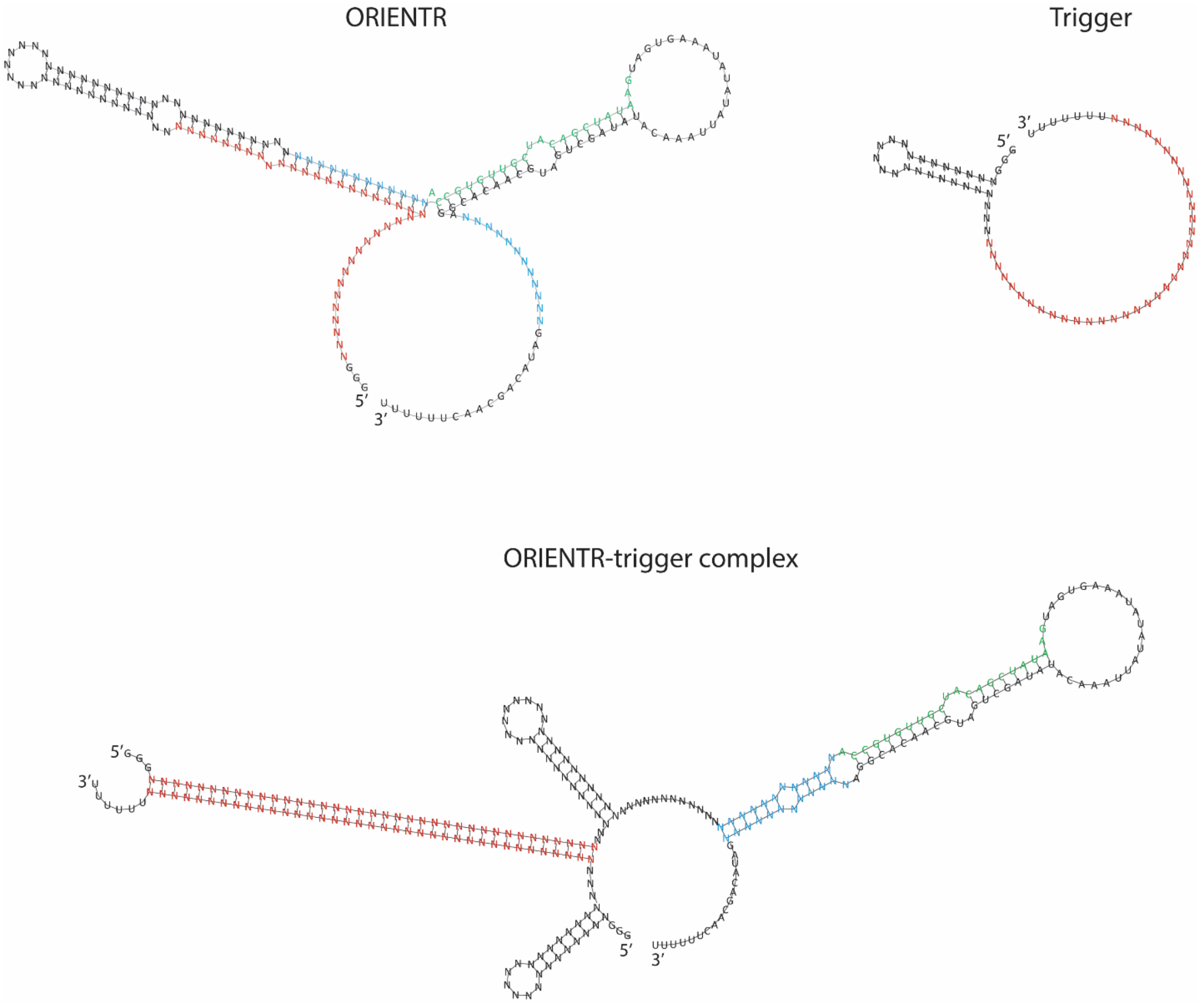

Figure S2 | NUPACK secondary structure design for the ORIENTR, trigger and ORIENTR-trigger complex. The red bases mark the trigger interaction domain, the blue bases mark the basal stem domain, and the green bases mark the amiRNA sequence.

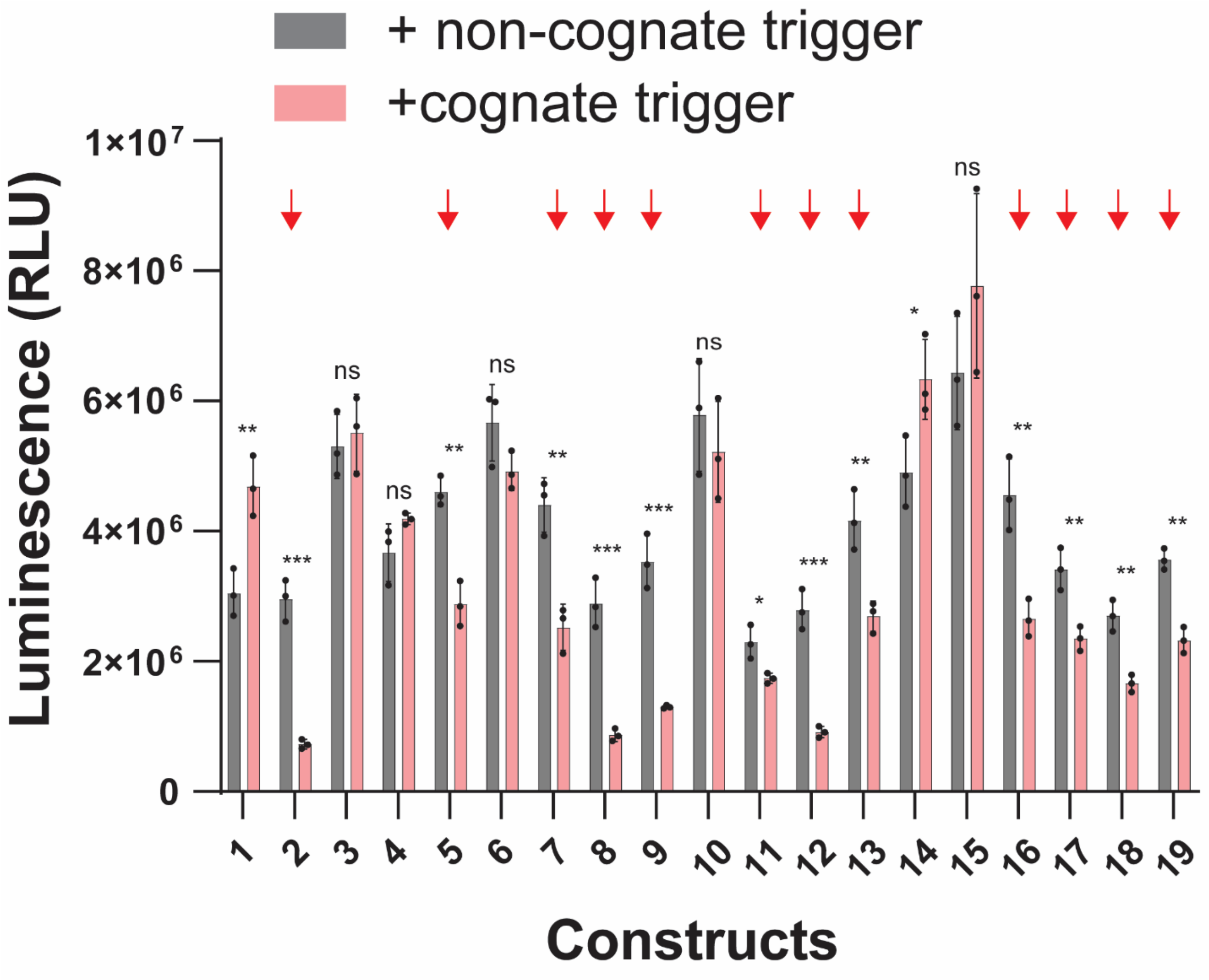

Figure S3 | Luciferase reporter signal for all 19 ORIENTR devices with non-cognate or cognate triggers. Red arrows indicate the constructs with significantly lower luminescence signal in response to the cognate trigger. (ns) $P > 0.05$, (∗) $P < 0.05$, (∗∗) $P < 0.01$, (∗∗∗) $P < 0.001$, unpaired two tailed *t* test, n=3 biological replicates, bars represent the mean ± s.d.

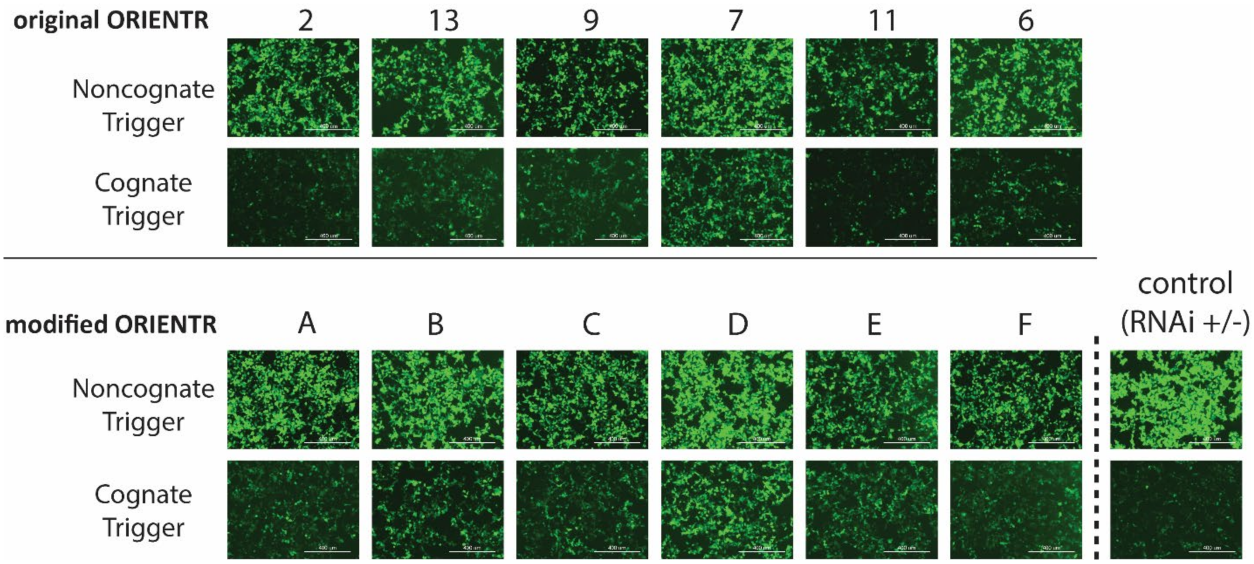

Figure S4 | Six ORIENTRs before and after modification with the leak-reduction motif in response to non-cognate/cognate triggers with a GFP reporter. RNAi positive control is from pri-miR-HSUR4; RNAi negative control is from pri-miR-16-2. n = 3 biological replicates. Scale bar represents 400 μm.

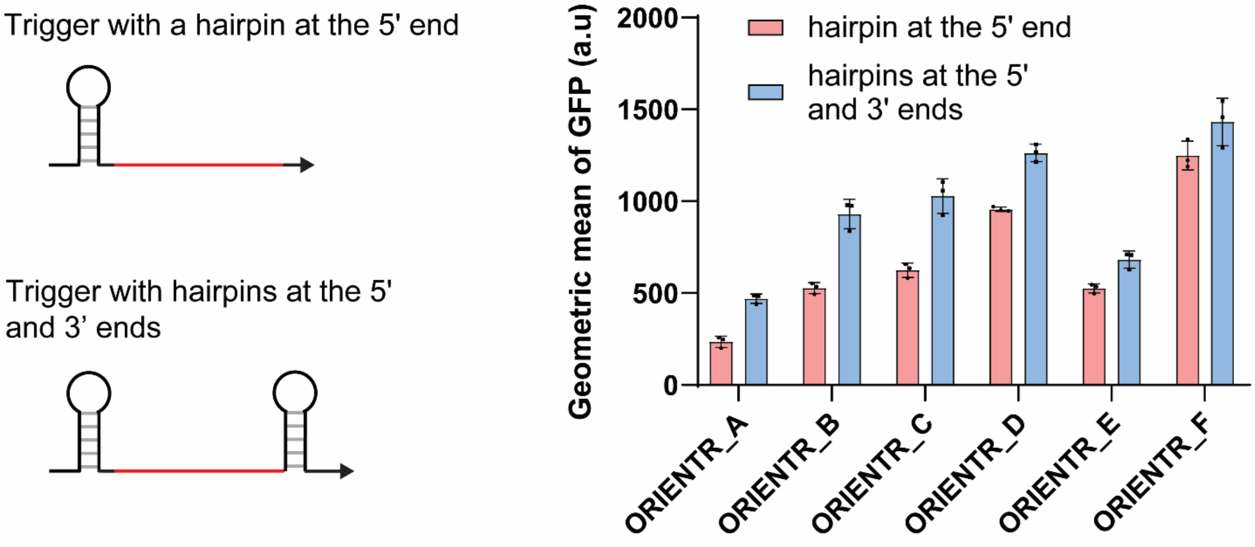

Figure S5 | Investigating the effect of 5' and 3' stem-loop structures on trigger RNA effectiveness. a, Secondary structure diagram for the RNA trigger with protecting hairpins on only the 5' end or on both the 5' and 3' ends. **b**, Comparison of GFP fluorescence across six ORIENTR devices with cognate triggers including either one or two protective hairpins. n = 3 replicates (cells from same passage number were used for 3 replicates), bars represent the mean ± s.d.

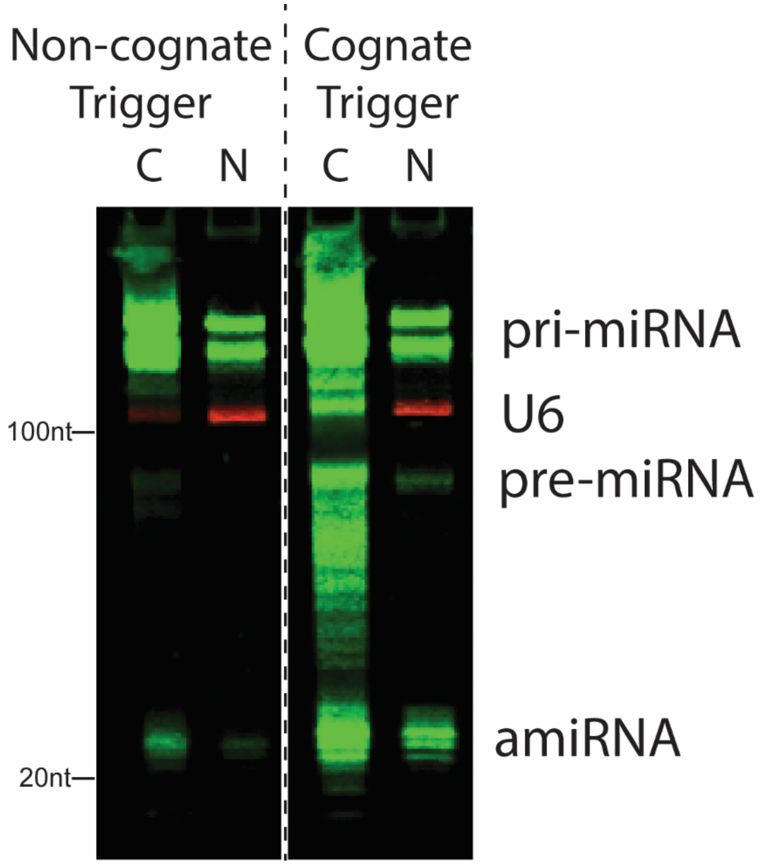

Figure S6 | Northern blot for ORIENTR_2 in response to non-cognate and cognate trigger RNAs. C denotes the cytoplasm; N denotes the nucleus. The amiR-HSUR4 probe was used to image pri-miRNA, pre-miRNA, and amiRNA localization. U6 RNA blotting was used as the control. n = 3 biological replicates.

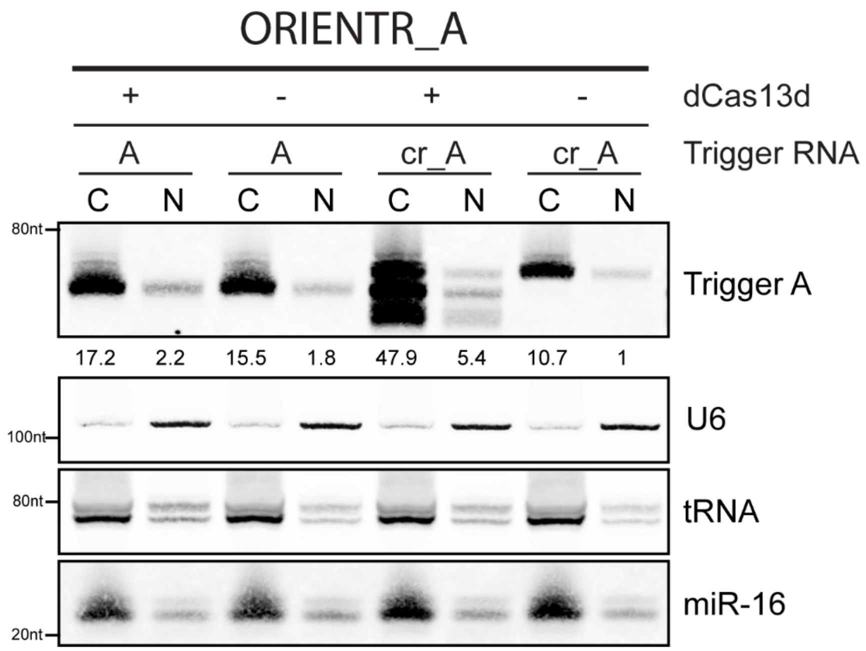

Figure S7 | Investigating trigger RNA cellular localization and abundance by northern blot. 'A' denotes trigger_A; 'cr_A' denotes trigger cr_A; 'C' denotes the cytoplasm; 'N' denotes the nucleus. U6 RNA, tRNA and miR-16 are used as nuclear or cytoplasmic RNA controls. n = 3 biological replicates.

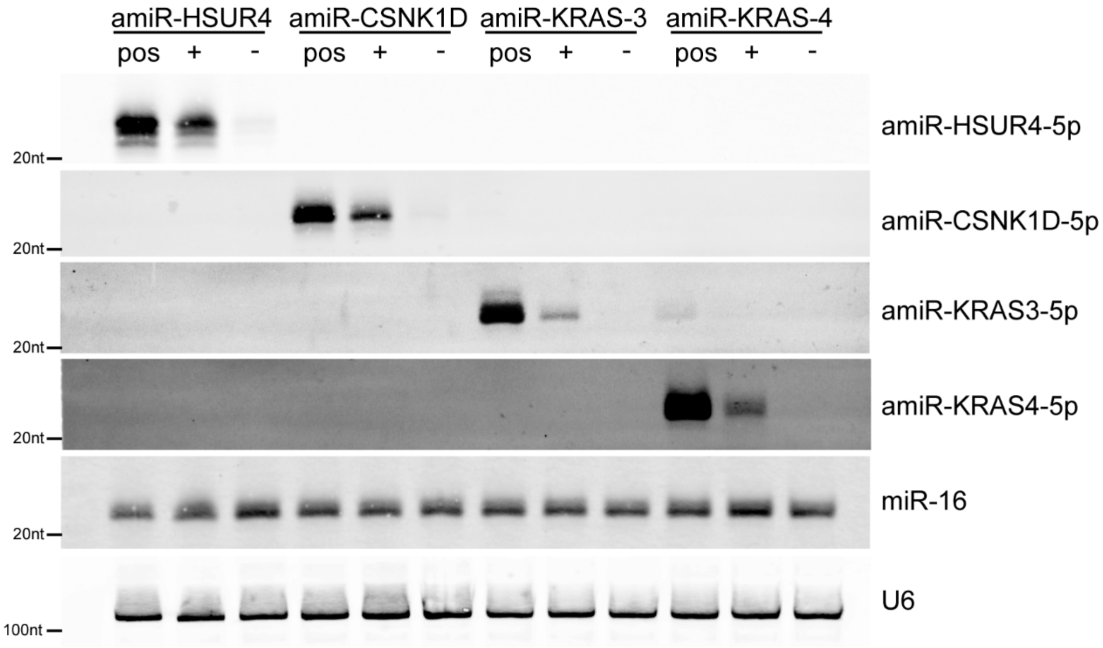

Figure S8 | Additional ORIENTRs targeting endogenous genes. The levels of U6 and miR-16 serve as loading controls. 'Pos' denotes positive control of pri-miR-HSUR4, pri-miR-CSNK1D, pri-miR-KRAS-3 pri-miR-KRAS-4, respectively. '+' denotes ORIENTR with cognate trigger. '-' denotes ORIENTR with non-cognate trigger. n = 3 biological replicates.

Supplementary Table S1. Plasmids for testing the pri-miRNA scaffold. Sequence information: the following sequences are inserted after a U6 promoter for transcription in cells.

Plasmid ID	Plasmide Name	Sequence
ZY-34	pri-miR-16_2	GCGCTAATACGACTCACTATAGGGATAAACATACATGCGCAACTGACATACTTGTTCCACTCTAGCAGCACGTAAATATTGGCGTAGTGAAATATATATTAAACACCAATATTACTGTGCTGCTTTAGTGTGACAGGGATACAGCAACTTTTTT
ZY-35	pri-miR-HSUR4	GCGCTAATACGACTCACTATAGGGATAAACATACATGCGCAACTGACATACTTGTTCCACTCACCGTGTTGCTACAGCTATAAGTAGTGAAATATATATTAAACATATAGCTGATGCAACACGGATAGTGTGACAGGGATACAGCAACTTTTTT

Supplemental Table S2. Plasmids for comparing amiRNA scaffolds. Sequence information: the following sequences are inserted after a chicken-beta-actin promoter and followed by an SV40 polyA signal sequence.

Plasmid ID	Plasmide Name	Sequence
YZ95	pCBA-pri-miR-16_2-HSUR4	ATAAACATACATGCGCAACTGACATACTTGTTCCACTCACCGTGTTGCTACAGCTATAAGTAGTGAAATATATATTAAACATATAGCTGATGCAACACGGATAGTGTGACAGGGATACAGCAACTGCTAGCCTTATAGCTGTAGCAACACGGTct
YZ349	pCBA-pri-miR-33_HSUR4	AGGGCTCTGCGTTTGCTCCAGGTAGTCCGCTGCTCCCTTGGGCCTGGGCCCACTGACAGCCCTGGTGCCTCTGGCCGGCTGCACACCTCCTGGCGGGCAGCTGTGACCGTGTTGCTACAGCTATAATGTTCTGGCAATACCTGTTATAGCTAAAACAACACGGTCACGGAGGCCTGCCCTGACTGCCCACGGTGCCGTGGCCAAAGAGGATCTAAGGGCACCGCTGAGGGCCTACCTAACCATCGTGGGGAATAAGGACAGTGTCACCCct
YZ341	pCBA-pri-miR-Ctrl	AGGGCTCTGCGTTTGCTCCAGGTAGTCCGCTGCTCCCTTGGGCCTGGGCCCACTGACAGCCCTGGTGCCTCTGGCCGGCTGCACACCTCCTGGCGGGCAGCGCAATACCTGTTATAGCTAAAACAACACGTCCACGGAGGCCTGCCCTGACTGCCCACGGTGCCGTGGCCAAAGAGGATCTAAGGGCACCGCTGAGGGCCTACCTAACCATCGTGGGGAATAAGGACAGTGTCACCCct

Supplementary Table S3. Plasmids for testing the pri-miRNA upstream flanking region sequence and structure requirements. Sequence information: the following sequences are inserted after a U6 promoter for transcription in cells.

Plasmid ID	Plasmide Name	Sequence
ZY-71	pri-miR-HSUR4_5'hpin	GGTCAGTTCGCGCTAATACGACTCACTATAGGGATAAACATACATGCGCGAACTGACATACTTGTTCCACTCACCGTGTTGCTACAGCTATAAGTAGTGAAATATATATTAAACATATAGCTGATGCAACACGGATAGTGTGACAGGGATACAGCAACTTTTTT
ZY-72	pri-miR-HSUR4_5'altseq	GCGCTAATACGACTCACTATAGGGATAAACATACATGCGCCCTCAACGATACTTGTTCCACTCACCGTGTTGCTACAGCTATAAGTAGTGAAATATATATTAAACATATAGCTGATGCAACACGGATAGTGTGACAGGGATACAGCAACTTTTTT
ZY-73	pri-miR-HSUR4_5'altseq+hpin	GCGTTGAGGGCGCTAATACGACTCACTATAGGGATAAACATACATGCGCCCTCAACGATACTTGTTCCACTCACCGTGTTGCTACAGCTATAAGTAGTGAAATATATATTAAACATATAGCTGATGCAACACGGATAGTGTGACAGGGATACAGCAACTTTTTT

Supplementary Table S4. Plasmids for testing the pri-miRNA basal stem region sequence and structure requirements. Sequence information: the following sequences are inserted after a U6 promoter for transcription in cells.

Plasmid ID	Plasmide Name	Sequence
ZY-35	pri-miR-HSUR4	GCGCTAATACGACTCACTATAGGGATAAACATACATGCGCAACTGACATACTTGTTCCACTCACCGTGTTGCTACAGCTATAAGTAGTGAAATATATATTAAACATATAGCTGATGCAACACGGATAGTGTGACAGGGATACAGCAACTTTTTT
ZY-129	basal_stem_n+m*	GCGCTAATACGACTCACTATAGGGATAAACATACATGCGCAACTGACATAAGCCATAACTCCACCGTGTTGCTACAGCTATAAGTAGTGAAATATATATTAAACATATAGCTGATGCAACACGGATAGTGTGACAGGGATACAGCAACTTTTTTT
ZY-130	basal_stem_m+n*	GCGCTAATACGACTCACTATAGGGATAAACATACATGCGCAACTGACATACTTGTTCCACTCACCGTGTTGCTACAGCTATAAGTAGTGAAATATATATTAAACATATAGCTGATGCAACACGGATGAGTCATGGCTGATACAGCAACTTTTTTT
ZY-131	basal_stem_n+n*	GCGCTAATACGACTCACTATAGGGATAAACATACATGCGCAACTGACATAAGCCATAACTCCACCGTGTTGCTACAGCTATAAGTAGTGAAATATATATTAAACATATAGCTGATGCAACACGGATGAGTCATGGCTGATACAGCAACTTTTTTT

Supplementary Table S5. Sequence for ORIENTRs and and their corresponding triggers. Sequence information: the following sequences are inserted after a U6 promoter for transcription in cells.

Plasmid ID	Plasmide Name	Sequence
ZY-132	ORIENTR_1	GGGTGAAATGAAATGAAAGCAGAGTGACAATAGAGGTAAGACGCAGTATCTGTATCCCGACAGATACTGCAACTTACCTCCATTGTCACTCTGCACCGTGTTGCTACAGCTATAAGTAGTGAAATATATATTAAACATATAGCTGATGCAACACGGAACAGAATGACAAGATACAGCAACTTTTTT
ZY-133	ORIENTR_2	GGGTGAGATGGATGTGATTGATAGGTAAGATTGTATTGCTCCTCGCCGACGCCTCCATCTGCGTCGGCGACAAGCAATACTATCTTACCTATCAACCGTGTTGCTACAGCTATAAGTAGTGAAATATATATTAAACATATAGCTGATGCAACACGGAGGATAAGTAAGAGATACAGCAACTTTTTT
ZY-134	ORIENTR_3	GGGTGATGGAGTAGAGAATGTATTAGGAGTTAGAATTAGACTACGAGGCTGCTGCTCGTCGCAGCCTCGTTTTCTAATTCTAACTCCTAATACAACCGTGTTGCTACAGCTATAAGTAGTGAAATATATATTAAACATATAGCTGATGCAACACGGAAGTATGAGGAGTGATACAGCAACTTTTTT
ZY-135	ORIENTR_4	GGGTGAATAAGAATGAAAGTATGGAAGATGTAGTCGTGTAACGGATATGCAACTGCGGCTTTGCATATCCTATACACGACAACATCTTCCATACACCGTGTTGCTACAGCTATAAGTAGTGAAATATATATTAAACATATAGCTGATGCAACACGGAATATGCAAGATGGATACAGCAACTTTTTT
ZY-151	ORIENTR_5	GGGTCGAATGTTAGTGTGCGCTGTTGAGGTATGTAGTTGATATTCCGGGCAGAAACATGACTGCCCGGAACCTCAACTACCTACCTCAACAGCGACCGTGTTGCTACAGCTATAAGTAGTGAAATATATATTAAACATATAGCTGATGCAACACGGAAGCTGGTGAGGTGATACAGCAACTTTTTT
ZY-152	ORIENTR_6	GGGTGATGAGAGTAAATGGACGAAATGTAGTAAGTAATGATATCTTCCATCCAAATCAAAGGATGGAAGACTTCATTACTAACTACATTTCGTCACCGTGTTGCTACAGCTATAAGTAGTGAAATATATATTAAACATATAGCTGATGCAACACGGACACGACATGTAGGATACAGCAACTTTTTT
ZY-153	ORIENTR_7	GGGTCGAATGGTAGTAAGTTAAGGATAATGTAATGTGTCTCACGAGGCTCTGTGGCTTACCAGAGCCTCGATAGACACATAACATTATCCTTAAACCGTGTTGCTACAGCTATAAGTAGTGAAATATATATTAAACATATAGCTGATGCAACACGGAATAAGAATAATGGATACAGCAACTTTTTT
ZY-154	ORIENTR_8	GGGTGAATGGCGTAAGTAGATGGTAGGATGACAGTTGAGTTCGCAGTGGAAACCTACTATTTTCCACTGCACACTCAACTCTCATCCTACCATCACCGTGTTGCTACAGCTATAAGTAGTGAAATATATATTAAACATATAGCTGATGCAACACGGAGATGGCAGGATGGATACAGCAACTTTTTT
ZY-155	ORIENTR_9	GGGTCGTTAGTTATTAGAGGTATGCTTAAGTTCGTATAGTCCTCGGTCTCGACCGGATCATCGAGACCGATTACTATACGCACTTAAGCATACCACCGTGTTGCTACAGCTATAAGTAGTGAAATATATATTAAACATATAGCTGATGCAACACGGAAGTATACTTAAGGATACAGCAACTTTTTT
ZY-156	ORIENTR_10	GGGTGAGAGCATAGAGACGTGAAGATGGGATAGGAAGTGACATGGCGTCTCTACGTTGTAAGAGACGCCACTTCACTTCCCATCCCATCTTCACACCGTGTTGCTACAGCTATAAGTAGTGAAATATATATTAAACATATAGCTGATGCAACACGGAATGAACATGGGAGATACAGCAACTTTTTT
ZY-157	ORIENTR_11	GGGTGATTAGTTGAAAGATATAGAAAGCGAAGTCGGTAAATCCGCGGTAAGCTGGTGAGGGCTTACCGCGTCTTTACCGAATTCGCTTTCTATAACCGTGTTGCTACAGCTATAAGTAGTGAAATATATATTAAACATATAGCTGATGCAACACGGACATAGCAAGCGAGATACAGCAACTTTTTT
ZY-158	ORIENTR_12	GGGTGCCCTGTCGTTCTAGTATGTAGCTTGTCGTGTTAGTGACTCTACCACTGGCTGGGTAGTGGTAGAGCGACTAACACTACAAGCTACATACACCGTGTTGCTACAGCTATAAGTAGTGAAATATATATTAAACATATAGCTGATGCAACACGGATTATGAAGCTTGGATACAGCAACTTTTTT

ZY-159	ORIENTR_13	GGGTCATTATGTTATTTCGCTAATCAGGCACGGTGTAGTTCCGCATAGCCCAACTTTCCGTGGGCTATGCATAACTACACAGTGCCTGATTAGCACCGTGTTGCTACAGCTATAAGTAGTGAAATATATATTAAACATATAGCTGATGCAACACGGATCTAAGCAGGCAGATACAGCAACTTTTTT
ZY-160	ORIENTR_14	GGGTGAGGGCAAATTGAGGCGCGTTTAGGCTGGTTAGATTATTGGCTGGAAATCAGCACATTTCCAGCCACGAATCTAACGAGCCTAAACGCGCACCGTGTTGCTACAGCTATAAGTAGTGAAATATATATTAAACATATAGCTGATGCAACACGGAACGCGGTTAGGCGATACAGCAACTTTTTT
ZY-162	ORIENTR_15	GGGTGATAATAGAATAATGGAGTAAACGAATGCGCTAGAACCCGGTTACTCATCTGAATGTGAGTAACCGACTTCTAGCGAATTCGTTTACTCCACCGTGTTGCTACAGCTATAAGTAGTGAAATATATATTAAACATATAGCTGATGCAACACGGACGAGTTAACGAAGATACAGCAACTTTTTT
ZY-163	ORIENTR_16	GGGTGCCTTTAGCTTGCAATGTCGTTTCTGTTCGTGTTAGAACGTGAAGCTTGCAGAACAAAGCTTCACGGCCTAACACGCACAGAAACGACATACCGTGTTGCTACAGCTATAAGTAGTGAAATATATATTAAACATATAGCTGATGCAACACGGACTGTCATTTCTGGATACAGCAACTTTTTT
ZY-164	ORIENTR_17	GGGTGATTAGATGACATACCCTTAACGAACCGGGATTTAGTCGGATGGCTCGACCGGATCCGAGCCATCCATCTAAATCCAGGTTCGTTAAGGGACCGTGTTGCTACAGCTATAAGTAGTGAAATATATATTAAACATATAGCTGATGCAACACGGAACCTTTACGAACGATACAGCAACTTTTTT
ZY-165	ORIENTR_18	GGGTCTCTACGCCGCTTCGAATGTTTATGTTATCGCTTGTAACCAGCGCTCCGTTTAAGTGGAGCGCTGGACACAAGCGACAACATAAACATTCACCGTGTTGCTACAGCTATAAGTAGTGAAATATATATTAAACATATAGCTGATGCAACACGGACAATGATTATGTGATACAGCAACTTTTTT
ZY-166	ORIENTR_19	GGGTCATATAGGTTAGGCAGCAGGTATCACAGTGTATCTCTGAGATGTATAGCCATAGCACTATACATCTATGAGATACAATGTGATACCTGCTACCGTGTTGCTACAGCTATAAGTAGTGAAATATATATTAAACATATAGCTGATGCAACACGGATGCAGCTATCACGATACAGCAACTTTTTT
ZY-140	trigger1	GGGCCCATAGGATTTTACCTATGGGAAACTTACCTCTATTGTCACTCTGCTTTCATTTCATTTCATTTTTT
ZY-141	trigger2 (trigger_A)	GGGCTCGATAGCGCAGACTATCGAGACAAGCAATACAATCTTACCTATCAATCACATCCATCTCATTTTTT
ZY-142	trigger3	GGGCACGTTCCTTAGTTGGAACGTGATTTCTAATTCTAACTCCTAATACATTCTCTACTCCATCATTTTTT
ZY-143	trigger4	GGGCCTAAGTTGAGAATAACTTAGGAATTACACGACTACATCTTCCATACTTTCATTCTTATTCATTTTTT
ZY-167	trigger5	GGGAGGTCGTGCAATAACACGACCTACTTCAACTACATACCTCAACAGCGCACACTAACATTCGATTTTTT
ZY-168	trigger6 (trigger_F)	GGGCTCAGCCAGCACTGTGGCTGAGCGTTCATTACTTACTACATTTCGTCCATTTACTCTCATCATTTTTT
ZY-169	trigger7 (trigger_D)	GGGCGCAGTTATTTGCTTAACTGCGGACAGACACATTACATTATCCTTAACTTACTACCATTCGATTTTTT
ZY-170	trigger8	GGGAGCCATGACCCTTATCATGGCTCTTACTCAACTGTCATCCTACCATCTACTTACGCCATTCATTTTTT
ZY-171	trigger9 (trigger_C)	GGGCCATAGATATTTCAATCTATGGAATACTATACGAACTTAAGCATACCTCTAATAACTAACGATTTTTT
ZY-172	trigger10	GGGCCTTTGGCATCCCTGCCAAAGGCATTCACTTCCTATCCCATCTTCACGTCTCTATGCTCTCATTTTTT
ZY-173	trigger11 (trigger_E)	GGGCCAACTCATAACTATGAGTTGGAACTTTACCGACTTCGCTTTCTATATCTTTCAACTAATCATTTTTT
ZY-174	trigger12	GGGACGGCACTGGGACAAGTGCCGTATAACTAACACGACAAGCTACATACTAGAACGACAGGGCATTTTTT
ZY-175	trigger13 (trigger_B)	GGGAGCCGACGACCGACCGTCGGCTAAGAACTACACCGTGCCTGATTAGCGAAATAACATAATGATTTTTT
ZY-176	trigger14	GGGACGTCTGCTACGCTGCAGACGTCTGAATCTAACCAGCCTAAACGCGCCTCAATTTGCCCTCATTTTTT
ZY-178	trigger15	GGGCACCAGAGCTGCGCCTCTGGTGATTTTCTAGCGCATTCGTTTACTCCATTATTCTATTATCATTTTTT
ZY-179	trigger16	GGGACCTGCGAACGAGGTCGCAGGTAAACTAACACGAACAGAAACGACATTGCAAGCTAAAGGCATTTTTT
ZY-180	trigger17	GGGTGCCGGTCTTATGCGACCGGCACATCTAAATCCCGGTTCGTTAAGGGTATGTCATCTAATCATTTTTT
ZY-181	trigger18	GGGTGCCCTCGCGGAGTCGAGGGCATGAACAAGCGATAACATAAACATTCGAAGCGGCGTAGAGATTTTTT
ZY-182	trigger19	GGGCCCTTACCTGTTTAGGTAAGGGACTGAGATACACTGTGATACCTGCTGCCTAACCTATATGATTTTTT
ZY-18	non-cognate trigger	GGGCCGTACTAAGGTGCTAGTACGGCATACTAACTCTACCTTACCTTCACTTCACTTCATTTTT

Supplementary Table S6. Design optimization for ORIENTR_2. Sequence information: the following sequences are inserted after a U6 promoter for transcription in cells.

Plasmid ID	Plasmide Name	Sequence
ZY-148	ORIENTR 2_1	GGGTGAGATGGATGTGATTGATAGGTAAGATTGTATTGCTCCTACAAGCAATACTATCTTACCTATCAACCGTGTTGCTACAGCTATAAGTAGTGAAATATATATTAAACATATAGCTGATGCAACACGGAGGATAAGTAAGAGATACAGCAACTTTTTT
ZY-149	ORIENTR 2_2	GGGTGAGATGGATGTGATTGATAGGTAAGATTGTATTGCTCTGATTTCACTCAGAGCAATACTATCTTACCTATCAACCGTGTTGCTACAGCTATAAGTAGTGAAATATATATTAAACATATAGCTGATGCAACACGGAGGATAAGTAAGAGATACAGCAACTTTTTT
ZY-150	ORIENTR 2_3	GGGTGAGATGGATGTGATTGATAGGTAAGATTGTATTGCTCTGATTTCACTAAAATCAGAGCAACCCAATCTTACCTATCAACCGTGTTGCTACAGCTATAAGTAGTGAAATATATATTAAACATATAGCTGATGCAACACGGAGGATAAGTAAGAGATACAGCAACTTTTTT
ZY-206	ORIENTR 2_4	GGGTGAGATGGATGTGATTGATAGGTAAGATTGTATTGCTCCTACAAGCAATACTATCTTACCTATCAACCGTGTTGCTACAGCTATAAGTAGTGAAATATATATTAAACATATAGCTGATGCAACACGGAGGATAAGTAAGAGATACAGCAACCTTATCATTTTTTT

Supplementary Table S7. ORIENTRs with the 3' stem loop motif. Sequence information: the following sequences are inserted after a U6 promoter for transcription in cells.

Plasmid ID	Plasmide Name	Sequence
ZY-206	ORIENTR_A (modified ORIENTR_2)	GGGTGAGATGGATGTGATTGATAGGTAAGATTGTATTGCTCCTACAAGCAATACTATCTTACCTATCAACCGTGTTGCTACAGCTATAAGTAGTGAAATATATATTAAACATATAGCTGATGCAACACGGAGGATAAGTAAGAGATACAGCAACCTTATCATTTTTTT
ZY-212	ORIENTR_B (modified ORIENTR_13)	GGGTCATTATGTTATTTCGCTAATCAGGCACGGTGTAGTTCCGCATAGCCCAACTTTCCGTGGGCTATGCATAACTACACAGTGCCTGATTAGCACCGTGTTGCTACAGCTATAAGTAGTGAAATATATATTAAACATATAGCTGATGCAACACGGATCTAAGCAGGCAGATACAGCAACGCTTAGTTTTTTT
ZY-202	ORIENTR_C (modified ORIENTR_9)	GGGTCGTTAGTTATTAGAGGTATGCTTAAGTTCGTATAGTCCTCGGTCTCGACCGGATCATCGAGACCGATTACTATACGCACTTAAGCATACCACCGTGTTGCTACAGCTATAAGTAGTGAAATATATATTAAACATATAGCTGATGCAACACGGAAGTATACTTAAGGATACAGCAACGTATACTATTTTTTT
ZY-208	ORIENTR_D (modified ORIENTR_7)	GGGTCGAATGGTAGTAAGTTAAGGATAATGTAATGTGTCTCACGAGGCTCTGTGGCTTACCAGAGCCTCGATAGACACATAACATTATCCTTAAACCGTGTTGCTACAGCTATAAGTAGTGAAATATATATTAAACATATAGCTGATGCAACACGGAATAAGAATAATGGATACAGCAACTTCTTAATTTTTTT
ZY-210	ORIENTR_E (modified ORIENTR_11)	GGGTGATTAGTTGAAAGATATAGAAAGCGAAGTCGGTAAATCCGCGGTAAGCTGGTGAGGGCTTACCGCGTCTTTACCGAATTCGCTTTCTATAACCGTGTTGCTACAGCTATAAGTAGTGAAATATATATTAAACATATAGCTGATGCAACACGGACATAGCAAGCGAGATACAGCAACTGCTATATTTTTTT
ZY-207	ORIENTR_F (modified ORIENTR_6)	GGGTGATGAGAGTAAATGGACGAAATGTAGTAAGTAATGATATCTTCCATCCAAATCAAAGGATGGAAGACTTCATTACTAACTACATTTCGTCACCGTGTTGCTACAGCTATAAGTAGTGAAATATATATTAAACATATAGCTGATGCAACACGGACACGACATGTAGGATACAGCAACTGTCGTATTTTTTT

Supplementary Table S8. Triggers with two hairpins. Sequence information: the following sequences are inserted after a U6 promoter for transcription in cells.

JL-ORIENTR1	uJL_trigger_A_hpin_5_3	GGGCTCGATAGCGCAGACTATCGAGACAAGCAATACAATCTTACCTATCAATCACATCCATCTCAGTTAATCGCCCACTTGATTAGCCGTTTTTTT

JL-ORIENTR2	uJL_trigger_B_hpin_5_3	GGGAGCCGACGACCGACCGTCGGCTAAGAACTACACCGTGCCTGATTAGCGAAATAACATAATGAGATCCCGCAATCTAGTGGGATCAACTTTTTT
JL-ORIENTR3	uJL_trigger_C_hpin_5_3	GGGCCATAGATATTTCAATCTATGGAATACTATACGAACTTAAGCATACCTCTAATAACTAACGATGCAGGCTGCACTGAGCTTGCACAATTTTTT
JL-ORIENTR4	uJL_trigger_D_hpin_5_3	GGGCGCAGTTATTTGCTTAACTGCGGACAGACACATTACATTATCCTTAACTTACTACCATTCGAGCTTCATCAACTAAGGTGAAGCAGTTTTTTT
JL-ORIENTR5	uJL_trigger_E_hpin_5_3	GGGCCAACTCATAACTATGAGTTGGAACTTTACCGACTTCGCTTTCTATATCTTTCAACTAATCAGCTCTTGCTCCTATGCGAGGGCAACTTTTTT
JL-ORIENTR6	uJL_trigger_F_hpin_5_3	GGGCTCAGCCAGCACTGTGGCTGAGCGTTCATTACTTACTACATTTCGTCCATTTACTCTCATCACACGTGGCAGGATAGTCACGTGCCCTTTTTT

Supplementary Table S9. Trigger sequence with the Cas13d crRNA scaffold. Sequence information: the following sequences are inserted after a U6 promoter for transcription in cells.

Plasmid ID	Plasmide Name	Sequence
ZY-248	cr_trigA	ggaaacccctaccaactggtcggggtttgaaacAGCAATACAATCTTACCTATCAATCACATCCATCTCATTTTTTT
ZY-250	cr_trigB	ggaaacccctaccaactggtcggggtttgaaacAACTACACCGTGCCTGATTAGCGAAATAACATAATGATTTTTTT

Supplementary Table S10. ORIENTRs for endogenous genes. Sequence information: the following sequences are inserted after a U6 promoter for transcription in cells.

Plasmid ID	Plasmide Name	Sequence
JL_ORIENTR_Hsp70	ORIENTR_Hsp70	GGGCCAGTATAAATTCATCTCTGCATGTAGAAAACGGAAAAAAAGCAAGTTCAGTACTTCACCTAAAAATTTCAACATTGCAAACACAGGAAATTGACGTGTTTTAGTTTCCTGTGTTTGCAACCGTGTTGCTACAGCTATAAGTAGTGAAATATATATTAAACATATAGCTGATGCAACACGGATGCAAACACAGCGGTCCCAAAAAGATACAGCAACGGGACCATTTTTT
RV7	pri-miR_KRAS_1	GCGCTAATACGACTCACTATAGGGATAAACATACATGCGCAACTGACATACTTGTTCCACTCaTTTCATACTggGTCTGCCTcGTAGTGAAATATATATTAAACaAGGCAGACaaAGTATGAAAaTAGTGTGACAGGGATACAGCAACTTTTTTT
RV8	pri-miR_KRAS_2	GCGCTAATACGACTCACTATAGGGATAAACATACATGCGCAACTGACATACTTGTTCCACTCaACTATAGGAcaTGATGCCTaGTAGTGAAATATATATTAAACaAGGCATCAacTCCTATAGTaTAGTGTGACAGGGATACAGCAACTTTTTTT
RV10	ORIENTR_KRAS_1	GGGTGAGATGGATGTGATTGATAGGTAAGATTGTATTGCTCCTACAAGCAATACTATCTTACCTATCAaTTTCATACTggGTCTGCCTcGTAGTGAAATATATATTAAACaAGGCAGACaaAGTATGAAAaGGATAAGTAAGAGATACAGCAACCTTATCATTTTTTT
RV11	ORIENTR_KRAS_2	GGGTGAGATGGATGTGATTGATAGGTAAGATTGTATTGCTCCTACAAGCAATACTATCTTACCTATCAaACTATAGGAcaTGATGCCTaGTAGTGAAATATATATTAAACaAGGCATCAacTCCTATAGTaGGATAAGTAAGAGATACAGCAACCTTATCATTTTTTT
RV1	pri-miR_CSNK1D	GCGCTAATACGACTCACTATAGGGATAAACATACATGCGCAACTGACATACTTGTTCCACTCtTAAAGCCATtgGTAACAGAcGTAGTGAAATATATATTAAACaTCTGTTACatATGGCTTTAtTAGTGTGACAGGGATACAGCAACTTTTTTT
RV2	pri-miR_KRAS_3	GCGCTAATACGACTCACTATAGGGATAAACATACATGCGCAACTGACATACTTGTTCCACTCtATTGTTGGAtcATATTCGTcGTAGTGAAATATATATTAAACaACGAATATatTCCAACAATtTAGTGTGACAGGGATACAGCAACTTTTTTT
RV3	pri-miR_KRAS_4	GCGCTAATACGACTCACTATAGGGATAAACATACATGCGCAACTGACATACTTGTTCCACTCcCAATTAGAAggTCTCAACTGGTAGTGAAATATATATTAAACaAGTTGAGAaaTTCTAATTGtTAGTGTGACAGGGATACAGCAACTTTTTTT
RV4	ORIENTR_CSNK1D	GGGTGAGATGGATGTGATTGATAGGTAAGATTGTATTGCTCCTACAAGCAATACTATCTTACCTATCAtTAAAGCCATtgGTAACAGAcGTAGTGAAATATATATTAAACaTCTGTTACatATGGCTTTAtGGATAAGTAAGAGATACAGCAACCTTATCATTTTTTT
RV5	ORIENTR_KRAS_3	GGGTGAGATGGATGTGATTGATAGGTAAGATTGTATTGCTCCTACAAGCAATACTATCTTACCTATCAtATTGTTGGAtcATATTCGTcGTAGTGAAATATATATTAAACaACGAATATatTCCAACAATtGGATAAGTAAGAGATACAGCAACCTTATCATTTTTTT
RV6	ORIENTR_KRAS_4	GGGTGAGATGGATGTGATTGATAGGTAAGATTGTATTGCTCCTACAAGCAATACTATCTTACCTATCAcCAATTAGAAggTCTCAACTgGTAGTGAAATATATATTAAACaAGTTGAGAaaTTCTAATTGtGGATAAGTAAGAGATACAGCAACCTTATCATTTTTTT

Supplementary Table S11. Additional sequence information for miRNA target sequences, Northern blot probes, and qPCR primers

Description	Sequence
miR-HSUR4-5p target site	TTATAGCTGTAGCAACACGGT
miR-HSUR4-5p sequence	ACCGUGUUGCUACAGCUAUAA
miR-HSUR4-5p northern probe sequence	TTATAGCTGTAGCAACACGGT/3AzideN/
miR-HSUR4-3p northern probe sequence	TCCGTGTTGCATCAGCTATAT/3AzideN/
miR-KRAS-1 5p northern probe	gAGGCAGACccAGTATGAAAt/3AzideN/
miR-KRAS-1 3p northern probe	tTTTCATACTttGTCTGCCTt/3AzideN/
miR-KRAS-2 5p northern probe	tAGGCATCAtgTCCTATAGTt/3AzideN/
miR-KRAS-2 3p northern probe	tACTATAGGAgtTGATGCCTt/3AzideN/
miR-KRAS-3 5p northern probe	GACGAATATGATCCAACAATA/3AzideN/
miR-KRAS-4 5p northern probe	CAGTTGAGACCTTCTAATTGG/3AzideN/
miR-CSNK1D 5p northern probe	GTCTGTTACCAATGGCTTTAA/3AzideN/
U6 northern probe sequence	GCAGGGGCCATGCTAATCTTCTCTGTATCG/3AzideN/
tRNA northern probe sequence	CTCATGCTCTACCGACTGAGCTAGCCGGGC/iAzideN/
noncognate trigger northern probe sequer	GTGAAGTGAAGGTAAGGTAGAG/3AzideN/
triggerA northern probe sequence	AGATGGATGTGATTGATAGGTA/iAzideN/
miR-16 northern probe sequence	CGC CAA TAT TTA CGT GCT GCT A/iAzideN/
miR-7 northern probe sequence	AACAACAAAATCACTAGTCTTCCA/3AzideN/
Kras-qPCR-F	CAGTAGACACAAAACAGGCTCAG
Kras-qPCR-R	TGTCGGATCTCtCTCACCAATG
GAPDH-qPCR-F	AATCCCATCACCATCTTCCA
GAPDH-qPCR-R	TGGACTCCACGACGTACTCA
