# Supplementary material for: Conditional RNA interference in mammalian cells via RNA transactivation
Source: Nat Commun. 2024 Aug 10;15:6855. doi: 10.1038/s41467-024-50600-w (PMC11316766; doi:10.1038/s41467-024-50600-w)
Supplement: Supplementary file 3 — Reporting Summary [file 41467_2024_50600_MOESM3_ESM.pdf]

nature portfolio

Corresponding author(s): Green, Xie

Last updated by author(s): 7/3/2024

Reporting Summary

Nature Portfolio wishes to improve the reproducibility of the work that we publish. This form provides structure for consistency and transparency in reporting. For further information on Nature Portfolio policies, see our Editorial Policies and the Editorial Policy Checklist.

Statistics

For all statistical analyses, confirm that the following items are present in the figure legend, table legend, main text, or Methods section.

n/a	Confirmed	
☐	☒	The exact sample size (*n*) for each experimental group/condition, given as a discrete number and unit of measurement
☐	☒	A statement on whether measurements were taken from distinct samples or whether the same sample was measured repeatedly
☐	☒	The statistical test(s) used AND whether they are one- or two-sided<br>*Only common tests should be described solely by name; describe more complex techniques in the Methods section.*
☒	☐	A description of all covariates tested
☒	☐	A description of any assumptions or corrections, such as tests of normality and adjustment for multiple comparisons
☐	☒	A full description of the statistical parameters including central tendency (e.g. means) or other basic estimates (e.g. regression coefficient) AND variation (e.g. standard deviation) or associated estimates of uncertainty (e.g. confidence intervals)
☐	☒	For null hypothesis testing, the test statistic (e.g. *F*, *t*, *r*) with confidence intervals, effect sizes, degrees of freedom and *P* value noted<br>*Give P values as exact values whenever suitable.*
☒	☐	For Bayesian analysis, information on the choice of priors and Markov chain Monte Carlo settings
☒	☐	For hierarchical and complex designs, identification of the appropriate level for tests and full reporting of outcomes
☒	☐	Estimates of effect sizes (e.g. Cohen's *d*, Pearson's *r*), indicating how they were calculated

Our web collection on statistics for biologists contains articles on many of the points above.

Software and code

Policy information about availability of computer code

Data collection	Northern blot images were obtained by Amersham Typhoon Scanner with ImageQuant TL (v7.0) software. GFP fluorescence images were obtained by Life Technologies EVOS FL cell imaging system (Thermo Fisher Scientific). Luciferase signal was read with a plate reader (BMG Labtech, CLARIOstar).
Data analysis	ImageQuant is used for analyzing irNorthern blot signals obtained from Amersham Typhoon scanner. GraphPad Prism8 is used for statistical analysis.

For manuscripts utilizing custom algorithms or software that are central to the research but not yet described in published literature, software must be made available to editors and reviewers. We strongly encourage code deposition in a community repository (e.g. GitHub). See the Nature Portfolio guidelines for submitting code & software for further information.

Data

Policy information about availability of data

All manuscripts must include a data availability statement. This statement should provide the following information, where applicable:

- Accession codes, unique identifiers, or web links for publicly available datasets
- A description of any restrictions on data availability
- For clinical datasets or third party data, please ensure that the statement adheres to our policy

The main data supporting the results in this study are available within the paper and the Supplementary Information. The datasets generated during and/or analyzed during the current study are available in the Source Data file provided with this paper.

Research involving human participants, their data, or biological material

Policy information about studies with human participants or human data. See also policy information about sex, gender (identity/presentation), and sexual orientation and race, ethnicity and racism.

Reporting on sex and gender	n/a
Reporting on race, ethnicity, or other socially relevant groupings	n/a
Population characteristics	n/a
Recruitment	n/a
Ethics oversight	n/a

Note that full information on the approval of the study protocol must also be provided in the manuscript.

Field-specific reporting

Please select the one below that is the best fit for your research. If you are not sure, read the appropriate sections before making your selection.

☒ Life sciences ☐ Behavioural & social sciences ☐ Ecological, evolutionary & environmental sciences

For a reference copy of the document with all sections, see nature.com/documents/nr-reporting-summary-flat.pdf

Life sciences study design

All studies must disclose on these points even when the disclosure is negative.

Sample size	No sample size calculation was done. Sample sizes were selected based on the magnitude of the effects observed and previous literature.
Data exclusions	No data were excluded.
Replication	All attempts at replication were successful for all experiments.
Randomization	Randomization is not relevant to this study.
Blinding	Blinding is not relevant to this study.

Reporting for specific materials, systems and methods

We require information from authors about some types of materials, experimental systems and methods used in many studies. Here, indicate whether each material, system or method listed is relevant to your study. If you are not sure if a list item applies to your research, read the appropriate section before selecting a response.

Materials & experimental systems

n/a	Involved in the study	
☐	☒	Antibodies
☐	☒	Eukaryotic cell lines
☒	☐	Palaeontology and archaeology
☒	☐	Animals and other organisms
☒	☐	Clinical data
☒	☐	Dual use research of concern
☒	☐	Plants

Methods

n/a	Involved in the study	
☒	☐	ChIP-seq
☒	☐	Flow cytometry
☒	☐	MRI-based neuroimaging

Antibodies

Antibodies used	Antibodies for KRAS (Thermo Fisher Scientific, 703345) and GAPDH (Sigma, G9545) were used for Western blots.
Validation	Anti-KRAS antibody validated in Senoo H, et al. KARATE: PKA-induced KRAS4B-RHOA-mTORC2 supercomplex phosphorylates AKT in insulin signaling and glucose homeostasis. Mol Cell. 2021 Nov 18;81(22):4622-4634.e8.<br>Anti-GAPDH antibody validated in Jeffrey G. Dickhout, et al. Integrated stress response modulates cellular redox state via induction of cystathionine γ-lyase: cross-talk between integrated stress response and thiol metabolism. J Biol Chem. 2012 Mar 2;287(10):7603-14.

Eukaryotic cell lines

Policy information about cell lines and Sex and Gender in Research

Cell line source(s)	HEK293T and Hela cells are from ATCC.
Authentication	None
Mycoplasma contamination	HEK293T and Hela cell tested mycoplasma negtive.
Commonly misidentified lines<br>(See ICLAC register)	None
